# Evaluation of Rosenmüller fossa depth and nasopharyngeal structures in individuals with cleft lip and palate using cone-beam computed tomography

**DOI:** 10.1186/s12903-026-08976-2

**Published:** 2026-06-20

**Authors:** Merva Güneyli, Emin Murat Canger

**Affiliations:** Department of Oral and Maxillofacial Radiology, Faculty of Dentistry, Erciyes University, Kayseri, Türkiye

**Keywords:** Cleft lip and palate, Cone-beam computed tomography, Eustachian tube, Rosenmüller’s fossa, Pharyngeal bursa

## Abstract

**Background:**

Cleft lip and palate (CLP) is a malformation traditionally attributed to muscle dysfunction but with deep anatomical origins. While early radiological signs of nasopharyngeal carcinoma often present as an asymmetric obliteration of the Rosenmüller fossa (RF), the impact of congenital CLP-related variations on this clinical detection remains a subject of debate with no definitive consensus. This study investigates these hypotheses by evaluating nasopharyngeal structural remodeling using cone-beam computed tomography (CBCT). To determine whether the morphometric changes are local or regional, unilateral (cleft/non-cleft sides), bilateral, and healthy individuals were compared. Based on our measurements, results indicate a specific structural transformation in the CLP nasopharynx characterized as a ‘narrow and long’ configuration, with both sides similarly affected even in unilateral cases. These findings suggest that CLP should be viewed not merely as a localized anatomical defect, but rather as a regional morphometric remodeling involving the entire nasopharyngeal complex.

**Methods:**

This retrospective study analyzed CBCT images of 78 CLP and 78 healthy individuals. Parameters included RF depth and type, torus levatorius (TL) length, inter-torus distance (ITD), posterior nasal spine - posterior pharyngeal wall distance (PNS-PPW), eustachian tube length (ETL), ET angle (ETA), tympanotubal angle (TTA), and pharyngeal bursa (PB) presence. Subjects were categorized as unilateral (cleft/non-cleft sides), bilateral, or control.

**Results:**

RF morphological types varied significantly between groups (*p* = 0.022), although RF depth (*p* = 0.155) and PB presence showed no significant differences. CLP groups exhibited significantly higher TTA and TL length (*p* < 0.001) and increased PNS-PPW distance, particularly in bilateral cases (*p* < 0.001). Conversely, ETL (*p* = 0.047) and ITD (*p* = 0.009) were significantly higher in controls. ETA showed no significant difference.

**Conclusions:**

CLP appears to be associated with morphometric remodeling of the nasopharynx and the eustachian tube, which is more pronounced in bilateral cases. These structural abnormalities may provide a potential framework for assessing velopharyngeal dysfunction and middle ear pathologies in this population.

## Introduction

Developmental disorders occurring during the early stages of embryogenesis, specifically between the 4th and 12th weeks, lead to failure in the fusion of facial prominences, resulting in orofacial anomalies such as cleft lip and palate (CLP). Unilateral cleft lip results from a failure of fusion between the maxillary and nasal prominences on one side, while bilateral cleft lip results from a failure on both sides. Inadequacy in the elevation, contact, or fusion of the palatal shelves is the primary cause of cleft palate [1]. Their prevalence is approximately 1.7/1000 live births, varying according to ethnic and geographic factors [2].

Cleft lip and palate is an anomaly associated with numerous clinical issues, including middle ear diseases and Eustachian tube (ET) dysfunction [1, 3, 4]. It has been demonstrated that cleft palate leads to shortening of the ET cartilage, thereby impairing normal tube function; furthermore, involvement of the tensor veli palatini (TVP) muscle restricts tube patency. Particularly in wide cleft palates, the ET is negatively affected as midface development is compromised [3]. This dysfunction is attributed to the abnormal positioning and hypoplasia of the levator veli palatini (LVP) and TVP muscles [5, 6]. This abnormal anatomy predisposes individuals to tube obstruction and a high incidence of middle ear infections [3, 7].

The nasopharynx is located behind the nasal cavity and above the soft palate. The Rosenmüller fossa (RF) is a bilateral pharyngeal recess in the nasopharynx that is of critical anatomical importance [8, 9]. Nasopharyngeal carcinoma (NPC), an epithelial carcinoma originating from the nasopharyngeal mucosa, frequently arises in this recess [10]. A recent study has shown that RF depth is greater in the CLP group and that morphological variations in this region may potentially mask the diagnosis of pathologies such as NPC [9]. However, there is still no complete consensus in the literature regarding the specific effects of cleft pathology on RF morphology. Therefore, a comprehensive evaluation of these structural variations is required to clarify their clinical significance. Furthermore, in childhood, the RF may undergo temporary obliteration due to pressure from adenoid tissue. These differences can lead to asymmetry [11, 12]. 

*The pharyngeal bursa (PB)* appears as an embryological epithelial remnant on the posterior wall of the nasopharynx; its size ranges from 5 to 10 mm, and its prevalence in the general population is below 7%. While mostly asymptomatic, a Tornwaldt cyst may develop if its opening becomes closed following surgical trauma (e.g., adenotonsillectomy) or inflammation. Although generally asymptomatic, large cysts can lead to complaints such as posterior nasopharyngeal discharge, occipital headache, halitosis, cervical muscle stiffness, and a feeling of fullness in the ear [13, 14].

To investigate the detailed anatomical structure and morphometric characteristics of the nasopharyngeal region, cone-beam computed tomography (CBCT) stands out as a reliable method due to its low radiation dose, high spatial resolution, and isotropic voxel structure [15, 16]. This morphological detailing capacity has established a solid foundation for the three-dimensional (3D) evaluation of nasopharyngeal structures in current literature.

The clinical significance of assessing RF morphology in individuals with CLP is multifaceted. Functionally, the RF is anatomically adjacent to the opening of the ET and the paratubal muscle system [17]. Since ET dysfunction and chronic middle ear diseases are significant complications in the CLP population [18], evaluating the RF is necessary to understand the broader structural abnormalities and spatial constraints occurring in the paratubal region. From a diagnostic perspective, the RF is also the primary site of origin for NPC [19, 20]. Since early-stage symptoms of NPC can mimic benign ear pathologies or TMJ disorders [17, 21, 22], clinical evaluation may be insufficient for early diagnosis. In this potential clinical diagnostic gap, radiological assessment is illuminating; the identification of subtle structural changes in the RF (such as obliteration, blunting, or asymmetry) is of paramount importance for early detection [21, 23, 24]. Furthermore, a recent study suggested that the RF is deeper in the CLP population, potentially causing the presence of NPC to be overlooked. Therefore, identifying the baseline three-dimensional anatomical variations of the RF in the CLP population is important. This knowledge not only sheds light on the structural basis of paratubal dysfunction but also helps prevent congenital anatomical variations from being misdiagnosed as pathology or masking early neoplastic changes during radiological and clinical evaluations.

In addition, the PB was included in our morphometric evaluation to ensure a comprehensive assessment of the nasopharyngeal region in individuals with CLP. Since the PB is an anatomical structure that can occasionally transform into a pathological entity known as a Tornwaldt cyst, a detailed examination is beneficial. In the context of the complex structural reorganization characteristic of CLP, establishing baseline morphological data for the PB, along with other nasopharyngeal structures, is valuable for preventing misinterpretations during accurate differential diagnosis and radiological follow-up.

However, previous CBCT studies evaluating the nasopharynx in individuals with CLP have primarily focused on overall airway volumes or isolated structural parameters. This study simultaneously evaluates eustachian tube (ET) parameters and specific nasopharyngeal structures (such as the Rosenmüller fossa, ITD, PNS-PPW, and pharyngeal bursa) in groups with unilateral CLP, bilateral CLP, and healthy controls, thereby contributing to the existing literature. Additionally, a detailed side-specific analysis was conducted to investigate the spatial distribution of the cleft’s anatomical effects. This design allows for the evaluation of different cleft types (unilateral and bilateral) to determine whether structural changes indicate localized defects or broader regional morphometric remodeling. The study group was limited to a highly homogeneous adolescent sample (ages 10–18) consisting solely of individuals with CLP who had a history of only primary palatoplasty. Through this methodology, the present study aims to provide clearer data regarding morphometric changes across the nasopharyngeal complex. In this study, we aimed to evaluate and compare the morphometric parameters of the nasopharyngeal region using CBCT, with a specific focus on the RF, which has been relatively under-addressed in the literature, between individuals with CLP and healthy controls, and to draw attention to structural variations in this region. We hypothesized that these parameters, particularly the RF, would differ significantly in the CLP population compared to healthy controls, and that the type of cleft (unilateral or bilateral) would be a determining factor in these changes.

## Materials and methods

This retrospective study was conducted using CBCT images from the archives of the Department of Oral and Maxillofacial Radiology. A total of 156 cases were included in the analysis, consisting of 78 individuals with CLP who met the inclusion criteria and 78 healthy controls matched to the study group by age and sex. The study protocol was approved by the Health Sciences Research Ethics Committee of the relevant university (Decision No: 2025/370) and conducted in accordance with the principles of the Declaration of Helsinki. Images of individuals aged 10–18 years, taken between January 2015 and May 2025, were evaluated. This age range was selected as it represents a group where nasopharyngeal structures can be reliably identified and the anatomical configuration following primary palatoplasty can be evaluated without the confounding influence of changes related to secondary surgical interventions [25, 26].

### Inclusion and Exclusion Criteria

Study Group: Patients aged 10–18 years with a diagnosis of CLP were included. All patients included in the study group had undergone routine primary soft-tissue palatoplasty during infancy. Patients with a history of any secondary interventions, such as alveolar bone grafting, pharyngeal flap surgery, or orthognathic procedures, were excluded from the study. The absence of such secondary surgeries was confirmed through a comprehensive review of the patients’ clinical records. Additionally, 3D CBCT scans were evaluated to confirm the presence of unrepaired bony clefts and to rule out radiological signs of secondary interventions (e.g., fixation devices).

Control Group: Absence of any history of systemic disease or craniofacial syndrome.

Common Exclusion Criteria: Presence of craniofacial syndromes, nasopharyngeal surgery/pathology history, trauma affecting alveolar bone integrity, and imaging artifacts.

### Grouping

The study group included 57 unilateral (36 male, 21 female) and 21 bilateral (13 male, 8 female) individuals with CLP. Data were divided into four side-based subgroups:

Group 1: Non-cleft side of unilateral CLP cases (*n* = 57 sides).

Group 2: Cleft side of unilateral CLP cases (*n* = 57 sides).

Group 3: Bilateral CLP cases (*n* = 42 sides).

Group 4: Control group (*n* = 156 sides).

### Imaging and standardization

Images were obtained using a single CBCT unit (NewTom 5G; Quantitative Radiology, Verona, Italy) and a standardized clinical protocol (110 kV, 3–5 mA, 0.25 mm voxel size). DICOM data were reconstructed using NNT software (v3.0). All evaluated CBCT scans had been previously acquired according to our institutional guidelines. As part of this routine protocol, which is inherently designed to minimize soft tissue variations, patients were scanned in the supine position with their heads properly secured. During the scan, patients were instructed to keep their teeth in maximum intercuspation, maintain a relaxed tongue posture, breathe calmly through their noses, and strictly avoid swallowing or any head movement. To prevent errors arising from head positioning, all data were realigned in standard planes relative to the maxillary plane and midline to ensure geometric accuracy.

All measurements were performed by a single radiologist with over 3 years of experience. Intra-observer reliability was assessed by re-evaluating 20 randomly selected cases with a two-week interval, and excellent agreement was observed (ICC = 0.91; 95% CI: 0.85–0.95).

### Morphometric evaluation

**Fig. 1:**
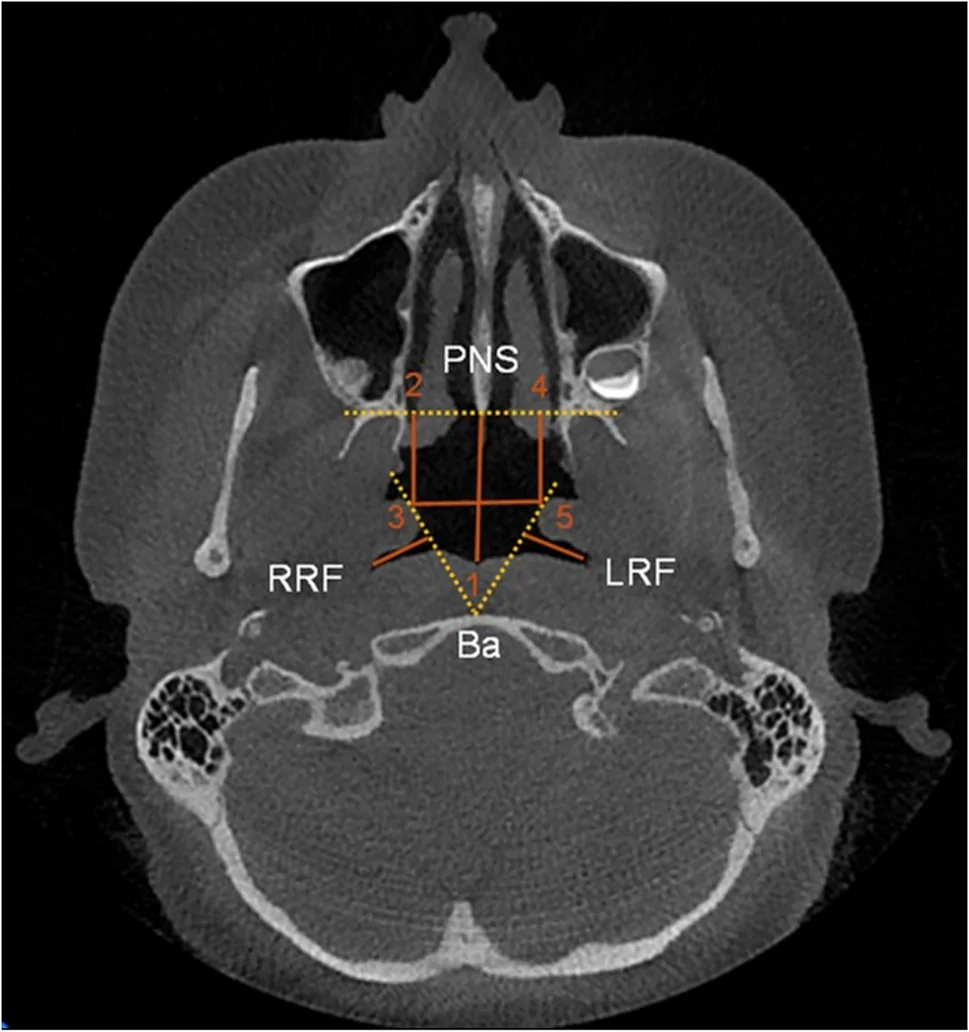
Morphometric analysis of nasopharyngeal structures and distances on an axial CBCT section. PNS posterior nasal spine, Ba basion, RRF right Rosenmüller fossa, LRF left Rosenmüller fossa, 1 posterior pharyngeal wall. PNS-PPW distance between the posterior nasal spine and the posterior pharyngeal wall; 2–3 right torus levatorius distance; 4–5 left torus levatorius distance; 3–5 inter-torus distance (distance between the right and left torus levatorius). Orange dashed lines indicate the reference planes used for anatomical orientation and standardization of the measurements

**Fig. 2:**
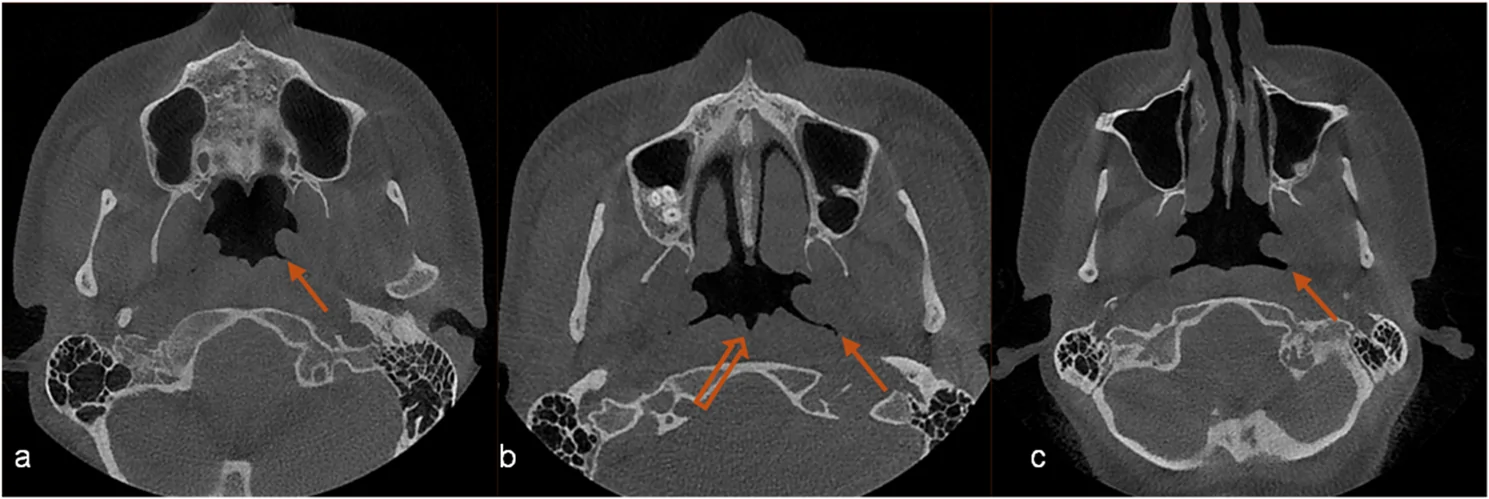
Axial CBCT images illustrating Rosenmüller fossa (RF) morphological types and the pharyngeal bursa. **(a)** Type A, **(b)** Type B, **(c)** Type C. In panel (**b**), the pharyngeal bursa located on the posterior pharyngeal wall is indicated by a hollow arrow. Straight arrows indicate the Rosenmüller fossa in all panels

Nasopharyngeal measurements were performed based on the reference system defined by Sutthiprapaporn et al. [27] using the posterior nasal spine (PNS) and basion (Ba) as reference points. Prior to the measurements, the PNS and Ba points were aligned as symmetrically as possible. Measurements were taken at the deepest and widest part of the Rosenmüller fossa (RF). Variables measured included right and left RF depths (RRF, LRF), torus levatorius distances (RTL, LTL), inter-torus distance (ITD), and the distance between the PNS and the posterior pharyngeal wall (PNS-PPW) [Fig. 1]. RF morphology was classified according to the reference system of Takasugi et al. [28] as Type A (< 5 mm), Type B (≥ 5 mm and < 1 mm opening) and Type C (≥ 5 mm and ≥ 1 mm opening) [Fig. 2].

Morphometric measurements of the ET were performed on CBCT multiplanar reconstruction (MPR) sections following the method defined by Duman et al. [29].

**Fig. 3:**
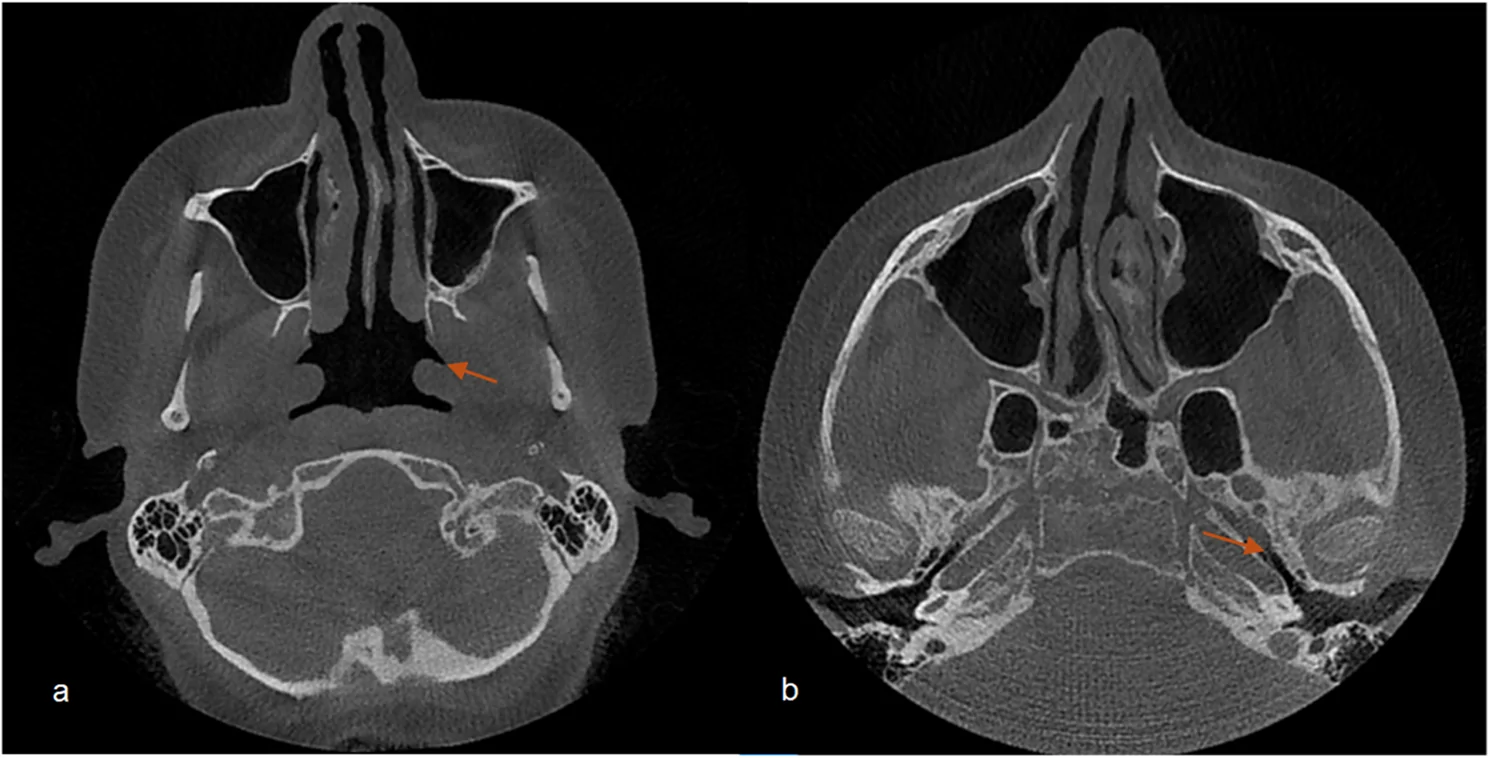
Axial CBCT sections demonstrating the Eustachian tube (ET) orifices. Orange arrows indicate the ET openings. **(a)** Pharyngeal orifice. **(b)** Tympanic orifice

The pharyngeal orifice was defined as the point of the ET lumen closest to the pharynx, and the tympanic orifice was defined as the lumen segment immediately preceding the appearance of the osseous external auditory canal [Fig. 3].

**Fig. 4:**
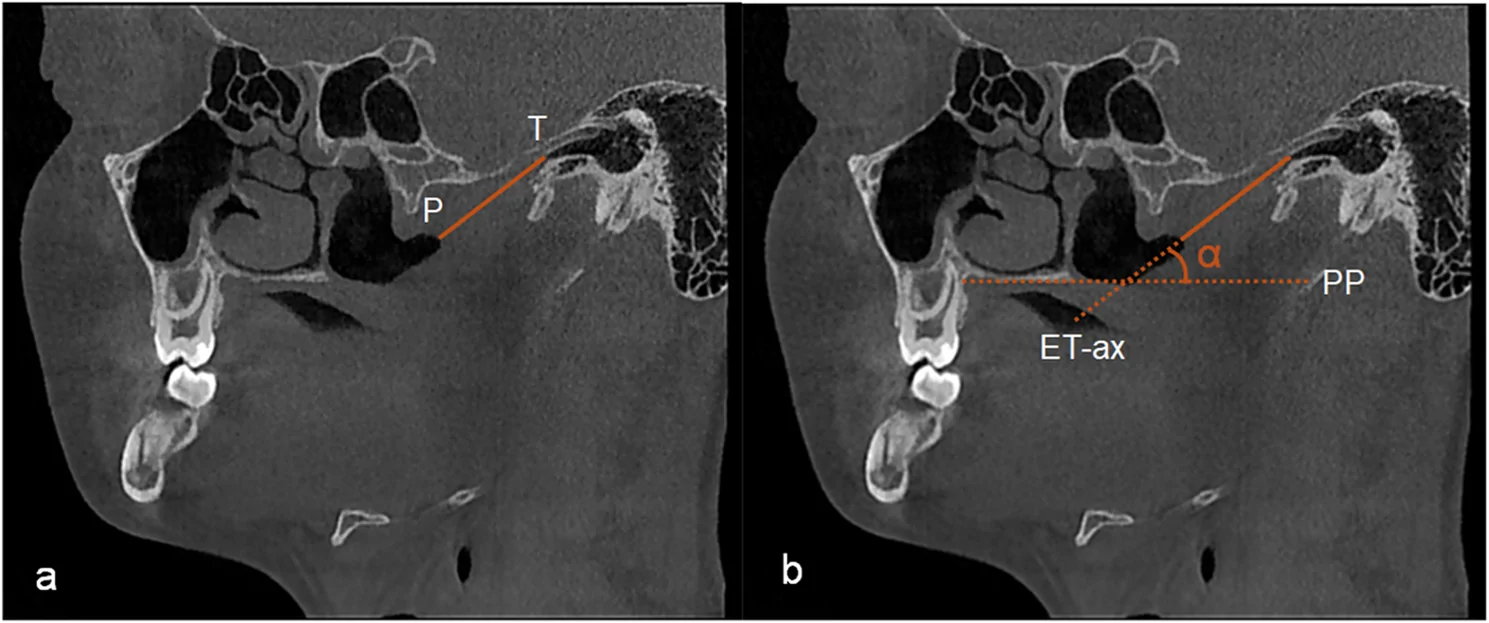
Morphometric analysis of the Eustachian tube (ET) on CBCT multiplanar reconstruction (MPR) images. (**a**) Measurement of the total ETL between points P and T. (**b**) Measurement of the ETA represented by the α symbol, measured between the PP and the ET-ax. P pharyngeal orifice, T tympanic orifice, PP palatal plane, ET-ax Eustachian tube long axis, ETL Eustachian tube length, ETA Eustachian tube angle

**Fig. 5:**
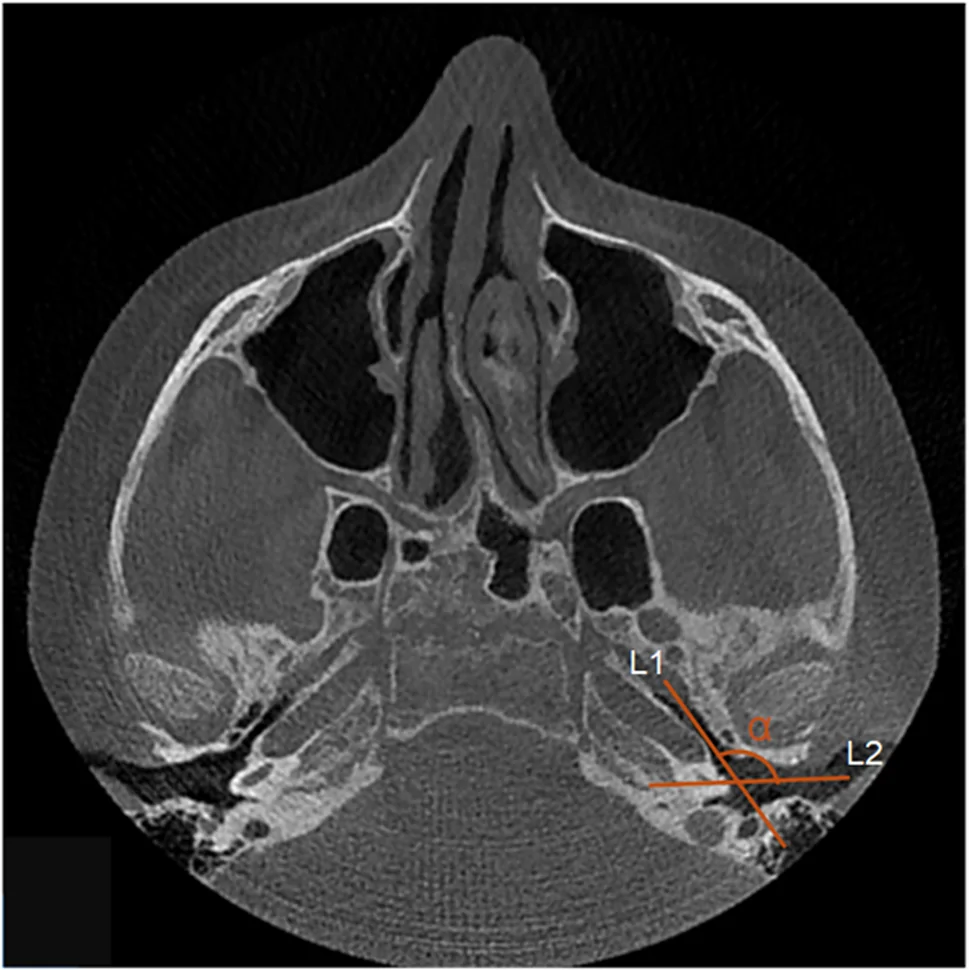
Demonstration of the tympanotubal angle (TTA) on an axial CBCT section. The TTA (α) was defined and measured as the angle between the axis of the osseous external auditory canal (indicated by line L1) and the long axis of the tympanic end of the Eustachian tube (indicated by line L2). L1 osseous external auditory canal axis, L2 Eustachian tube long axis (tympanic end), α tympanotubal angle

- The Eustachian tube length (ETL) was calculated as the linear distance between these two orifices.
- The Eustachian tube angle (ETA) was measured as the angle between a reference line parallel to the palatal plane and a line along the anatomical long axis of the ET [Fig. 4].

- The tympanotubal angle (TTA) was calculated as the angle between the long axis of the tympanic end of the ET and the axis of the osseous external auditory canal [Fig. 5] [29–31].

An a priori power analysis was conducted using the G*Power software (version 3.1.9.7; Heinrich-Heine-Universität, Düsseldorf, Germany) to determine the required sample size. To detect significant differences in continuous morphometric variables between groups, assuming a moderate effect size (Cohen’s d = 0.45), a 0.05 alpha error probability, and 80% statistical power (1 – *β* error), the minimum required sample size was calculated as 78 individuals per group. Consequently, a total of 156 participants (78 in the CLP group and 78 in the control group) were included in the study to meet these statistical criteria [32].

Statistical analyses were conducted using IBM SPSS Statistics 26.0, with a significance level set at *p* < 0.05. The conformity of continuous variables to a normal distribution was evaluated using the Shapiro–Wilk test. Normally distributed variables were compared using the independent samples t-test (for two groups) or one-way analysis of variance (ANOVA) (for more than two groups). Following ANOVA, Tukey HSD or Games–Howell post-hoc tests were applied based on the homogeneity of variances assessed by Levene’s test. Categorical variables (such as RF type and PB presence) were analyzed using the Pearson chi-square test. To assess potential lateral asymmetries, bilateral anatomical structures were analyzed separately for each side (unit-based analysis). Conversely, parameters located anatomically on the midline or where the bilateral relationship is represented by a single quantitative value (such as ITD and PNS-PPW) were evaluated as a single measurement per individual. The analyzed measurement units were as follows: Group 1 (unilateral CLP, non-cleft side): 57 units; Group 2 (unilateral CLP, cleft side): 57 units; Group 3 (bilateral CLP): 42 units; and Group 4 (control group): 156 units. To control for potential intra-subject dependency between these bilateral measurements, a subject-based sensitivity analysis was performed alongside the unit-based approach.

## Results

A total of 78 individuals with CLP (57 unilateral and 21 bilateral) and 78 individuals from a healthy control group were analyzed. The demographic data of the groups are presented in (Table 1 ).

**Table 1:** Comparison of sex and age distribution among groups

		Group				Test	*p*
		Group 1	Group 2	Group 3	Group 4		
		*n* (%)	*n* (%)	*n* (%)	*n* (%)		
Sex^**^	Male	36 (63.2%)	36 (63.2%)	26 (61.9%)	98 (62.8%)	0.021	0.999
	Female	21 (36.8%)	21 (36.8%)	16 (38.1%)	58 (37.2%)		
Age (mean ± SD)^*^		14.04 ± 2.71	14 ± 2.71	13.95 ± 2.9	14 ± 2.78	0.007	0.999

^*^= One-way ANOVA test

^**^= Pearson chi-square test SD: Standard deviation, n: Number of sides

No significant difference in mean ages was found between the groups (*p* = 0.999). Gender distributions were also similar, and no statistically significant difference was detected between the groups (*p* = 0.999)

When RF morphological types were evaluated, Type A was the most frequently observed type across all groups (Table 2). It was determined that the distribution of RF types showed a statistically significant difference between the groups (Chi-square = 14.840; *p* = 0.022). No statistically significant difference was found between the groups in terms of the presence of the pharyngeal bursa (PB) (*p* = 0.092).

**Table 2:** Comparison of Rosenmüller fossa morphological types and pharyngeal bursa distribution among groups

		Group				Chi-square	*p*	Post-hoc
		Group 1	Group 2	Group 3	Group 4			
		*n* (%)	*n* (%)	*n* (%)	*n* (%)			
RF Type^a^	Type A	46 (80.7%)	46 (80.7%)	30 (71.4%)	107 (68.6%)	14.840	0.022	1–4
	Type B	2 (3.5%)	2 (3.5%)	0 (0%)	0 (0%)			2–4
	Type C	9 (15.8%)	9 (15.8%)	12 (28.6%)	49 (31.4%)			
PB^a^	Absent	42 (73.7%)	42 (73.7%)	24 (57.1%)	94 (60.3%)	6.431	0.092	
	Present	15 (26.3%)	15 (26.3%)	18 (42.9%)	62 (39.7%)			

*RF* Rosenmüller fossa, *PB* Pharyngeal bursa, *n* Number of sides

^a^ Pearson chi-square test

Post-hoc: Inter-group multiple comparisons based on the Pearson chi-square test. Significant differences are indicated in the corresponding column at the (*p* < 0.05) level

No significant difference was found between the groups regarding RF depth measurements (*F* = 1.759; *p* = 0.155) (Table 3).

TL length showed a significant difference between the groups (*F* = 13.350; *p* < 0.001), and in multiple comparisons, the means of Group 1, Group 2, and Group 3 were significantly higher than Group 4.

Eustachian tube length showed a significant difference between the groups (*F* = 2.678; *p* = 0.047), and in multiple comparisons, the mean of Group 4 was higher than Group 2.

No significant difference was found between the groups regarding Eustachian tube angle values (*F* = 1.459; *p* = 0.226).

Tympanotubal angle values showed a significant difference between the groups (*F* = 9.271; *p* < 0.001), and in multiple comparisons, the means of Group 1, Group 2, and Group 3 were higher than Group 4.

The ITD showed a significant difference between the groups (*F* = 3.962; *p* = 0.009), and in multiple comparisons, the mean of Group 4 was higher than Group 1 and Group 2. (In unilateral CLP cases, this measurement anatomically represents a single value per individual).

The PNS-PPW distance showed a significant difference between the groups (*F* = 32.460; *p* < 0.001). In multiple comparisons, the mean of Group 3 was higher than Group 1, Group 2, and Group 4; additionally, the means of Group 1 and Group 2 were higher than Group 4. (In unilateral CLP cases, this measurement anatomically represents a single value per individual).

**Table 3:** Comparison of morphometric measurements among groups

	Group				F	*p*	Post-hoc
	Group 1	Group 2	Group 3	Group 4			
(mean ± SD)	(mean ± SD)	(mean ± SD)	(mean ± SD)				
RF	2.61 ± 3.78	2.65 ± 3.66	3.16 ± 3.48	3.73 ± 4.13	1.759	0.155	
TL	19.26 ± 4.83^a^	19.24 ± 4.88^a^	20.42 ± 7.01^a^	16.42 ± 3.22^b^	13.350	< 0.001	1,2,3 > 4
ETL	25.91 ± 4.04	25.53 ± 3.75^a^	25.69 ± 3.72	26.88 ± 3.3^b^	2.678	0.047	2 < 4
ETA	33.38 ± 9.76	34.33 ± 9.49	33.84 ± 8.78	32 ± 6.18	1.459	0.226	
TTA	144.97 ± 9.75^a^	144.56 ± 9.08^a^	146.17 ± 7.74^a^	139.38 ± 9.38^b^	9.271	< 0.001	1,2,3 > 4
ITD	19.82 ± 3.33^a^	19.82 ± 3.33^a^	20.56 ± 3.24	21.74 ± 5.55^b^	3.962	0.009	1,2 < 4
PNS-PPW	22.97 ± 6.49^b^	22.97 ± 6.49^b^	26.36 ± 6.07^a^	18.57 ± 3.45^c^	32.460	< 0.001	3 > 1,2,4; 1,2 > 4

RF: Rosenmüller fossa depth, TL: Torus levatorius length, ETL: Eustachian tube length, ETA: Eustachian tube angle, TTA: Tympanotubal angle, ITD: Inter-torus distance, PNS-PPW: Distance between the posterior nasal spine and the posterior pharyngeal wall. mean: Mean, SD: Standard deviation (For midline parameters (ITD and PNS-PPW), measurements represent a single value per subject; therefore, the analysis for these variables was conducted based on the total number of individuals (*n* = 78 CLP and *n* = 78 control) rather than the number of sides.)

One-way ANOVA test. Post-hoc: Inter-group comparisons were performed using Tukey HSD or Games-Howell tests. Groups with different superscript letters (^a, b,c^) in the same row are statistically significantly different at the (*p* < 0.05) level

### Linear regression

In the multiple linear regression analysis, it was determined that the TL (*β* = −0.181; *p* < 0.001) and ETL (*β* = −0.080; *p* = 0.015) variables exerted significant negative effects on RF depth; conversely, ITD (*β* = 0.199; *p* < 0.001), PNS-PPW (*β* = 0.202; *p* < 0.001), and Type C morphological variables (*β* = 0.662; *p* < 0.001) were found to have significant positive effects. The model demonstrated a remarkably high explanatory power (*R*^*2*^ = 0.737) (Table 4).

As a result of the sensitivity analysis, it was confirmed that the significant inter-group differences observed in the TTA, TL, PNS-PPW, and ITD parameters were fully consistent across both models. For parameters such as ETL and ETA, statistical significance remained at a marginal level (*p* < 0.10) in the subject-based analysis due to the reduction in sample size; however, the general orientation of these findings was found to be parallel to the results of the unit-based analysis (*p* < 0.05).

**Table 4:** Multiple linear regression analysis of the relationships between Rosenmüller fossa and other morphometric parameters

Dependent variable	Independent variable	β	t	*p*	*R* ^2^
RF	TL	-0.181^*^	-4.159	< 0.001	0.737
	ETL	-0.080^*^	-2.437	0.015	
	ETA	0.011	0.346	0.730	
	TTA	0.026	0.804	0.422	
	ITD	0.199^*^	5.254	< 0.001	
	PNS-PPW	0.202^*^	4.584	< 0.001	
	Type B	0.034	1.059	0.291	
	Type C	0.662^*^	16.843	< 0.001	
	PB	-0.032	-1.015	0.311	

*RF* Rosenmüller fossa depth, *TL* Torus levatorius length, *ETL* Eustachian tube length, *ETA* Eustachian tube angle, *TTA* Tympanotubal angle, *ITD* Inter-torus distance, *PNS-PPW* Anteroposterior distance between the posterior nasal spine and the posterior pharyngeal wall, *Type B/C* Rosenmüller fossa morphological type classification, *PB* Presence of pharyngeal bursa

^*^ Statistically significant at the (*p* < 0.05) level

## Discussion

In this study, the morphometric architecture of the nasopharyngeal complex in individuals with CLP was analyzed in depth via CBCT. Although RF depth, PB prevalence, and ETA showed no significant differences, our results revealed a structural remodeling specific to the CLP population, particularly in terms of RF morphological type, ETL, TL length, TTA, PNS-PPW, and ITD distance.

Statistical differences were detected in the distribution of RF morphological types between the groups (*p* = 0.022). Although Type A was the most frequently observed morphology across all groups, this tendency was more pronounced in the CLP group compared to the control group. Conversely, the more frequent observation of Type C morphology in the control group suggests that the RF tends to be anatomically ‘shallower’ in CLP patients. These findings are generally consistent with previous radiological studies focusing on RF depth and nasopharyngeal airway dimensions [28, 33]. While our observations align with the established patterns of RF morphology, the divergence from the Type B-predominant distribution reported in some studies [34] may be attributed to variations in craniofacial morphology and the distinct resolution capacities of the imaging modalities utilized (e.g., CBCT vs. MDCT). From a clinical perspective, the ‘shallower’ structure observed in CLP patients may facilitate the visualization of landmarks during endoscopic procedures [28].

The statistical similarity observed between the groups regarding the presence of the *pharyngeal bursa* (*p* = 0.092) suggests that this anatomical variation is related to individual developmental processes and population characteristics rather than CLP pathology. Our data are consistent with previous findings that reported no correlation with PB presence in patients with unilateral CLP [9], further indicating that CLP does not have a significant effect on bursa formation. Furthermore, the similarity between the PB rates detected in our control group and those reported in the literature for the healthy population confirms the biological consistency of our findings and their alignment with established data [28, 33].

Although there was no significant difference between the groups in absolute RF depth (*p* = 0.155), regression analysis confirmed its positive correlation with nasopharyngeal volume and neighboring structures (PNS-PPW, ITD). This finding is consistent with previous studies conducted in healthy populations that reported a positive correlation between RF depth and PNS-PPW distance [12]. While some studies have reported that the RF may be deeper in children with CLP [9], others have documented variations in RF morphology due to different imaging methods such as MDCT [34, 35]. Our findings suggest that the shallow RF morphology is not an individual finding; rather, it is part of a broader geometric shift associated with nasopharyngeal volume and developmental adaptations [25, 26], which may contribute to a more accessible anatomical space during clinical evaluations [28]. In this context, CBCT provides a three-dimensional mapping of surrounding tissues. Therefore, it may serve as a useful complementary tool in preoperative evaluation by providing objective evidence of variations that are difficult to detect via physical examination.

Torus Levatorius length, a critical component of nasopharyngeal morphometry, was found to be significantly longer in the CLP groups compared to the control group in our study. Since TL anatomically represents the mucosal projection of the LVP muscle, this increase in length is considered a reflection of deviations in the muscle’s anatomical vector or the mucosal remodeling process secondary to the cleft anatomy. Although TVP plays the primary role in the active opening of the Eustachian tube, it should not be overlooked that this structural difference in the LVP muscle may have indirect effects on the supportive functional dynamics of the tube [36, 37]. Furthermore, our findings are consistent with the positive correlation reported between TL length and RF depth [12], supporting the close interrelation of anatomical structures within the nasopharynx.

The fact that the ITD in the control group was significantly wider compared to CLP patients (*p* = 0.009) indicates a developmental transverse narrowing of the nasopharynx. Although the ITD values in the literature show heterogeneity due to methodological differences and imaging methods [12, 34], the findings of our control group are consistent with the reported spectrum. Regression analysis revealed a strong positive correlation between ITD and RF depth (*β* = 0.199; *p* < 0.001), demonstrating the structural integrity between the transverse width of the nasopharynx and the anatomical depth of the fossa. In this context, it is thought that the ITD narrowing observed in individuals with CLP is a structural adjustment that occurs simultaneously with a decrease in RF depth. This narrowing may alter the anatomical vectors of the paratubal muscles, potentially leading to eustachian tube dysfunction (ETD) [38]. As reported in the literature, rapid maxillary expansion (RME) in children with CLP improves hearing loss and otitis media by increasing nasopharyngeal volume [39]. In this context, our finding of significant ITD narrowing in patients with CLP is consistent with the restricted transverse dimension data reported for this population in the literature [12, 34]. Furthermore, since all CLP patients in our study had a history of primary palatoplasty, this transverse narrowing may be attributed to the combined effects of intrinsic anatomical alterations inherent to the cleft and the restrictive nature of secondary scar tissue resulting from early surgical repair.

When Eustachian tube parameters were examined, our findings revealed that individuals with CLP exhibit significantly shorter ETL (*p* = 0.047) and wider TTA (*p* < 0.001) compared to healthy controls, consistent with previous literature [4, 29, 40, 41]. Although the ETA did not show a statistically significant inter-group difference, the lower ETA values observed in the CLP population suggest that the tube tends to maintain a more horizontal orientation relative to the skull base [29, 37, 40]. Our data suggest that the age-related verticalization process expected during adolescence [36, 42] may be impaired in the CLP population, with a trend toward a more horizontal orientation of the tube. It has been reported in the literature that shorter ETL and lower ETA values, as well as wider TTA, significantly reduce ventilation efficiency and disrupt drainage mechanisms, potentially contributing to the structural basis for the pathogenesis of chronic otitis media [4, 37, 38]. In particular, it is emphasized that the increase in TTA may facilitate the spread of upper respiratory tract infections to the middle ear, thereby increasing susceptibility to otitis media [4]. In addition, our findings revealed that the ETL is shorter on the cleft side than on the opposite side in unilateral CLP cases (*p* < 0.05), which suggests a potential influence of local developmental asymmetry on tube morphometry. Conversely, the lack of statistical asymmetry in all other nasopharyngeal measurements (PNS-PPW, ITD, RF depth; *p* > 0.05) suggests that CLP is not merely a local anatomical defect, but a generalized developmental remodeling affecting the entire nasopharyngeal complex.

These findings emphasize that, even in unilateral cases, the contralateral side warrants careful evaluation for similar anatomical risks. Consequently, our data support the idea that the Eustachian tube in the CLP population possesses different anatomical characteristics. As documented in the literature [4, 37, 38], it should be considered in clinical practice that these anatomical features may predispose individuals to middle ear problems.

In our study, the PNS-PPW distance was found to be significantly higher in the CLP group, particularly in bilateral cases, compared to healthy controls (*p* < 0.001). The alignment of the PNS-PPW values detected in our control group (18.57 mm) with the healthy population data reported in the literature [12, 34] (17.7 mm and 19.92 mm, respectively) confirms the validity of our methodology and control group. On the other hand, the marked increase in PNS-PPW distance observed in CLP patients, particularly in bilateral cases, reflects a substantial expansion in the sagittal dimension of the nasopharynx. The simultaneous observation of this sagittal increase in the PNS-PPW distance with the transverse narrowing in the ITD distance suggests that the nasopharynx in individuals with CLP does not just undergo a volumetric change, but structurally assumes a distinct, elongated, and constricted geometric pattern. This morphometric reshaping indicates that developmental defects in the palate and pharyngeal muscular system force the surrounding bone and soft tissue framework into this specific geometry. Additionally, the fact that the patients have undergone primary palatoplasty is an important factor that should be considered when evaluating this anatomical remodeling.

In the regression analysis, the positive correlation detected between PNS-PPW and RF depth (*β* = 0.202; *p* < 0.001), supports the existing findings showing that the anteroposterior extension of the nasopharynx tends to increase fossa depth [12]. Furthermore, although it has been reported that the PNS-PPW distance may be related to age and gender [43], the matching of groups in our study in terms of these variables strengthens the finding that the detected differences are related to the cleft pathology. Another noteworthy finding is that no asymmetry was detected in the morphometric parameters between the cleft side and the healthy side in unilateral CLP cases (*p* > 0.05).

Our findings suggest that the nasopharynx and ET complex in individuals with CLP possess a specific morphometric characterization that deviates significantly from the healthy population. As noted above, these findings indicate a pronounced geometric shift characterized by sagittal elongation and transverse constriction. The wider TTA and shorter ETL are key components of this structural deformity, and as noted in the literature, such anatomical variations are considered risk factors for reduced ventilation and drainage efficiency.

Furthermore, the altered nasopharyngeal morphology in the CLP population may provide an anatomical framework that accompanies the well-documented muscular dysfunctions. Our study, which specifically included patients who had not undergone secondary surgical interventions, suggests that these anatomical deviations associated with cleft pathology are a developmental outcome; however, the modifying effects of primary surgical scars on growth must also be acknowledged. To assess these alterations, CBCT-based morphometry serves as a valuable complementary tool in clinical evaluation by objectively mapping complex three-dimensional structural details.

There are some methodological limitations to this study. The retrospective design may harbor a risk of selection bias. Furthermore, the measurements were performed by a single observer; although intra-observer agreement was high, inter-observer variation could not be evaluated. Additionally, our study is entirely a morphometric analysis and was not correlated with ET function (tympanometry, etc.). Treating the right and left measurements in bilateral cases as independent units (unit of analysis: side) may carry a risk of pseudoreplication; however, sensitivity analyses showed consistent results on both unit and subject bases. Another limitation is that focusing on the 10–18 age range may limit the generalization of results to very early childhood or adult populations. Additionally, within this specific adolescent period, the potential volumetric variations of adenoid tissue were not quantitatively assessed. Because adenoid hypertrophy can narrow the nasopharyngeal space and temporarily alter Rosenmüller fossa morphology, it should be considered a potential confounding factor. Finally, it should be noted that while patients with secondary surgical interventions were strictly excluded, all individuals in the CLP group had undergone primary soft-tissue palatoplasty during infancy. As is inherent to such clinical populations, the potential influence of early surgical scarring and tissue tension on the long-term growth and morphometric remodeling of the nasopharynx cannot be completely ruled out.

In conclusion, this CBCT-based morphometric study has revealed that the nasopharyngeal architecture in individuals with CLP significantly differs compared to healthy individuals. The widespread morphometric deviations observed across the structural parameters highlight a distinct geometric remodeling specific to the CLP population, rather than isolated dimensional changes.

These structural alterations, which become more pronounced particularly in bilateral cases, suggest a potential relationship between the severity of the cleft pathology and anatomical disruption.

The fact that nasopharyngeal structures are symmetrically affected even in unilateral cases indicates that CLP may be associated with a generalized developmental remodeling process affecting the entire nasopharyngeal complex. This situation emphasizes that both sides must be evaluated with the same meticulousness during clinical evaluation. Ultimately, CBCT-based three-dimensional mapping provides a valuable and objective dataset for recognizing these anatomical risk factors specific to the CLP population and for developing clinical management strategies.

## Data Availability

The datasets generated during and/or analyzed during the current study are available from the corresponding author on reasonable request.
